# Regional difference in multi-psychotropic drug prescription in Japan and its associated factors: an ecological study using national health insurance claims data

**DOI:** 10.1007/s43999-022-00018-y

**Published:** 2023-01-05

**Authors:** Tasuku Okui, Naoki Nakashima

**Affiliations:** Medical Information Center, Kyushu University Hospital, Maidashi3-1-1 Higashi-Ku, Fukuoka City Fukuoka Prefecture, 812-8582 Japan

**Keywords:** Psychotropic drugs, Prescriptions, Japan, Geography, Polypharmacy

## Abstract

**Background:**

In Japan, regulations preventing the prescriptions of various types of psychotropic drugs have become stricter in recent years. However, the areas where multi-psychotropic drug prescriptions are common and the geographic factors that contribute to the regional difference, have not been studied. In this study, we used data from all claims in Japan to examine regional differences in the prescription for multi-psychotropic drugs using an ecological study.

**Methods:**

The National Database of Health Insurance Claims and Specific Health Checkups of Japan Open data in 2019 were used. The outcome was the number of prescriptions for four or more different types of anxiolytics and hypnotics as well as for three or more of the same kind of psychotropic drugs (any one of anxiolytics, hypnotics, antipsychotics, antidepressants) for outpatients in each area. Among the 335 secondary medical areas in Japan, the data on 331 areas were used in the analysis. The standardized claim ratio (SCR), an indicator of the number of this multi-psychotropic drug prescription, correcting for regional differences in distribution of population by age and sex, was calculated for each of the secondary medical areas. The spatial cluster detection technique was used to locate a cluster of high-SCR areas. Furthermore, factors associated with regional differences in the SCRs were examined by a spatial statistics model.

**Results:**

North Japanese regions tend to have high SCRs, and 13 areas in Hokkaido were identified as the most likely cluster (cluster with the highest likelihood ratio) for multi-psychotropic drug prescription. Furthermore, a spatial regression analysis revealed that the proportion of people with lower educational levels, the total number of prescriptions per capita, and the number of physicians working in psychiatric departments per capita were statistically positively associated with the SCR for the prescription of multiple psychotropic drugs.

**Conclusions:**

It was suggested that socioeconomic and medical characteristics of areas are related to the regional variation in the multi-psychotropic drug prescriptions, however, further research using individual-level data is required to confirm these results.

## Background

Polypharmacy is one of the most frightening public health problems worldwide [1, 2]. In Japan over the past few years, the prevalence of polypharmacy has decreased [3, 4], while the prevalence of potentially inappropriate medications has increased [3]. It is well known that multiple psychotropic drugs tend to be prescribed concurrently in Japan [5, 6], and psychotropic drugs tend to be associated with potentially inappropriate medications [7]. It is known that polypharmacy of psychotropic drugs causes negative drug effects, such as fall and impaired cognitive function [8–10]. In response to this issue, medical service fee revisions on multi-psychotropic drug prescriptions were implemented from 2012 to 2018 in Japan [11–13]. In Japan, “the Healthcare bill check and payment organization” checks claims data and pays medical costs directly to the medical facilities instead of the health insurers in the health insurance system. Medical facilities submit claims data and demands medical costs against “the Healthcare bill check and payment organization” and the medical institutions are subsequently reimbursed. When multiple types of psychotropic medications (three or more anxiolytics, three or more hypnotics, four or more antipsychotics, or four or more antidepressants) were prescribed, it was decided to reduce the prescription and drug fees that are reimbursed in 2012 and 2014, and the regulation tightened up until the revision in 2018 [11]. Specifically, when three or more anxiolytics, three or more hypnotics, three or more antipsychotics, three or more antidepressants, or four or more anxiolytics and hypnotics were prescribed at one time, the prescription and drug fees that a medical institution could obtain started to be reduced from the medical fee service revision in 2018. In other words, fees that medical institutions can claim for their medical practices decrease as a punishment when the multi-psychotropic drug prescription is conducted. Three or more types of drugs means three or more types of drug substances (generic names) and fee reduction is applied when multiple drugs are prescribed at a time (in one prescription). In contrast, fees for the prescription and medication are not reduced when psychotropic medications are prescribed temporarily or when antipsychotics or antidepressants are unavoidably prescribed by a doctor with sufficient training in psychiatric clinical practice. As a result, the proportion of multi-psychotropic drug prescriptions decreased over the years in Japan [12, 13].

Identification of patient traits or geographic areas linked to the multi-psychotropic drug prescriptions is crucial for furthering the improvement of multi-drug prescriptions. Some studies examined regional differences in the frequency of depression consultations or the number of psychotropic medications prescribed in Japan [14, 15]. On the other hand, there are no studies that looked into regional differences in multi-drug prescriptions of psychotropic drugs in Japan. Furthermore, it is crucial to detect factors associated with the regional difference because it will help in taking preventive measures for the prescriptions in the high-risk regions. Particularly, few studies examined an association between polypharmacy and areal or individual socioeconomic status in Japan. In addition, no studies have looked into the relationship between multi-psychotropic drug prescriptions and local socioeconomic indicators. In other countries, regional socioeconomic status is known to be related to polypharmacy or psychotropic drug prescriptions [16, 17].

In this study, we used nationally representative health insurance claims data to reveal regional differences in the multi-drug prescriptions of psychotropic drugs in Japan and to identify factors related to the prescriptions.

## Methods

### Outcome variable

In this study, regional-level data was used to conduct an ecological study. Specifically, aggregate data from Japan’s National Database of Health Insurance Claims and Specific Health Checkups of Japan (NDB) were used [18]. NDB is a national database created by the Ministry of Health, Labour, and Welfare in Japan [19], and it compiles claims data nationally. Notably, “the Healthcare bill check and payment organization” submits claims data to the Ministry of Health, Labour, and Welfare. Electronic receipt data from hospitals, medical clinics, dental clinics, and pharmacies are included in the NDB, but paper receipt data are not included. In Japan, it is known that over 95% of receipts are electronic receipts [20]. Additionally, data on medical expenses that are not covered by insurance and uninsured persons’ data are not included in the NDB data [21]. Numerous studies have been carried out using the NDB’s aggregate data which are publicly accessible as open data [14, 22, 23].

As were mentioned before, when three or more anxiolytics, three or more hypnotics, three or more antipsychotics, three or more antidepressants, or four or more anxiolytics and hypnotics were prescribed at one time, reduction of the prescription and drug fees occurs from 2018. Data on the number of this prescription of multiple psychotropic drugs are available in the open data of NDB, and the number was used as the outcome in this study. In this study, “multi-psychotropic drug prescription” refers to the prescription of three or more types of psychotropic drugs or four or more types of anxiolytics and hypnotics at a time (in one prescription). Data on the number of multi-psychotropic drug prescription by sex and age group, as well as the number of multi-psychotropic drug prescriptions by secondary medical area in 2019, were used. The number of prescriptions was available, but the number of individuals prescribed was not available in the data. It is also possible that an individual receives multiple multi-drug prescriptions in a year.

A secondary medical area is an area unit that includes several municipalities in a prefecture. It is an area unit bigger than a municipality but smaller than a prefecture in Japan. The secondary medical area was designed by the Ministry of Health, Labour, and Welfare to provide sufficient medical care in that region [24]. There were 335 secondary medical areas in 2019 in Japan; the prescription data for four areas were not available in the data. In the NDB Open data, information was not available if there were fewer than 10 medical practices in a region of fewer than 3 medical institutions that housed those practices. In addition, data on out-of-hospital prescriptions were used in this study. Data on in-hospital prescriptions were not used in this study because there were many areas where the number of in-hospital prescriptions for multi-drug psychotropic drugs was not available.

### Explanatory variables

Demographic, medical, and socioeconomic characteristics in areas were used for investigating an association with multi- psychotropic drug prescription. Specifically, population density, proportion of single-person households, proportion of divorced persons, total number of prescriptions per capita, number of physicians per capita, number of physicians working in a psychiatric department per capita, number of medical institutions with a psychiatric department per capita, unemployment rate, and proportion of persons with lower educational level were used. Data on out-of-hospital prescriptions were used for the number of total prescriptions, similar to the number of multi-psychotropic drug prescriptions. The medical institutions include hospitals and medical clinics. The proportion of people with a lower educational level refers to the proportion of people who have graduated from elementary school or junior high school among population aged 15 or more, and the unemployment rate is the number of unemployed people per labor force population. The proportion of divorced persons refers to the number of divorced people per population aged 15 years or more. Data on the number in each secondary medical area were available for each characteristic, and the proportion of persons or number per capita was calculated by dividing the number by population. In addition, age group- and area-specific data were available for the proportion of people with a lower educational level, the unemployment rate, and the proportion of divorced persons. Therefore, age-standardized values were calculated for those variables using the overall population in Japan as the standard population. All the information was collected from the government statistics, and data on the Census, the Municipalities Area Statistics of Japan, the Survey of Medical Institutions, the Statistics of Physicians, dentists, and Pharmacists, and the population, demographics, and household numbers based on the basic resident register were used [25–29]. Data on population density and the total number of prescriptions per capita in 2019 were used, whereas data in 2020 were used for the other characteristics because those data were not available in 2019. Furthermore, map data for each of the secondary medical areas were obtained from the digital national land information published by the Ministry of Land, Infrastructure, Transport, and Tourism [30].

### Statistical analysis

The age distribution and sex ratio differ between secondary medical areas, and the standardized claim ratio (SCR) was calculated for each area, as performed in previous studies [14, 22, 31]. The rate of multi-psychotropic drug prescription (Number of prescriptions per population) for each age group and sex in Japan was calculated. The expected number of multi-psychotropic drug prescriptions for each combination of age group, sex, and secondary medical area was calculated by multiplying the population and the rate in all of Japan. By summing the expected numbers for all the age groups and sexes, the expected number of the multi-psychotropic drug prescription for each area was calculated. The SCR was calculated by dividing the actual number of prescriptions by the expected number. SCR is an indirect standardization method, similar to the standardized mortality ratio. By mapping the SCR, the geographical difference in the SCR by area was demonstrated. Furthermore, an adjacency matrix among areas was made from the map. An adjacency matrix is a matrix indicating whether two regions are contiguous or not and is used in the spatial statistical model.

A spatial cluster detection method was also used to detect a spatial cluster of high-SCR areas, that is, the areas where preventive measures against multi-psychotropic drug prescriptions are particularly needed. Specifically, a flexible spatial scan statistic using a restricted likelihood ratio was used [32, 33]. The method can detect a cluster of areas that are not necessarily circular in shape. In the method, the number of multi-psychotropic drug prescriptions was assumed to follow a Poisson distribution. A most likely cluster, which maximizes the likelihood ratio most, and secondary clusters were detected [34–38]. Simply put, the most likely cluster is the areas whose values are most different from the other areas among any area combination. The values as well as the number of multi-psychotropic drug prescriptions in a cluster are taken into account to determine the most likely cluster and secondary clusters in the method. Because high value area clusters can be assessed from a result showing geographic difference in the SCR, only the top 3 clusters (the most likely cluster, secondary cluster 1, and secondary cluster 2) were detected by the method as conducted in previous studies [37, 38]. A hypothesis test for investigating whether an observed number of multi-psychotropic drug prescriptions in the cluster is equal to the expected number was constructed, and the p-value was calculated using Monte Carlo simulation.

An ecological study was performed for examining the relationship between SCR and municipal characteristics. The correlation coefficient between the SCR and each of the municipal characteristics was calculated. Furthermore, multivariate regression analysis was used for investigating the association. To account for spatial autocorrelation of the SCR among areas, a spatial regression model with the conditional autoregressive error was used [39]. All the explanatory variables were standardized, and for each variable, the standardized partial regression coefficient (SPRC), its 95% confidence intervals, and p-value were calculated. Statistical tests were two-sided in the regression analysis, and a p-value less than 0.05 was defined as statistically significant. The ecological study used data from 317 areas after 14 areas that were not adjacent to the other areas were excluded because a spatial adjacency was needed in the spatial analysis.

All statistical analyses were carried out using R, ver. 4.1.3 (https://www.R-project.org/).

## Results

Table 1 depicts the number of multi-psychotropic drug prescriptions and the number per 100,000 persons by age group and sex in all of Japan. In particular, the number of prescriptions per 100,000 people was high in 40–59 years, and it was around 2,000 for those age groups. In addition, the number of prescriptions per 100,000 people for women was higher compared to that for men in 15–89 years.

**Table 1:** Number of multi-psychotropic drug prescriptions and the number per 100,000 persons by age group and sex in all of Japan

	Men		Women	
Age group	Number of the prescriptions	Number of the prescriptions per 100,000 persons	Number of the prescriptions	Number of the prescriptions per 100,000 persons
0–4	437	17.3	343	14.3
5–9	926	33.9	746	28.7
10–14	2,163	76.6	1,296	48.2
15–19	5,041	165.8	6,713	232.1
20–24	10,193	309.2	17,233	551.5
25–29	20,001	604.2	33,775	1081.0
30–34	34,284	937.0	51,842	1484.1
35–39	54,036	1349.9	71,157	1845.0
40–44	76,112	1634.6	95,126	2115.9
45–49	96,911	1944.8	113,579	2342.6
50–54	87,938	2058.4	94,644	2250.3
55–59	70,645	1843.5	77,805	2031.5
60–64	49,359	1316.4	58,202	1521.6
65–69	42,126	948.8	55,373	1173.8
70–74	39,113	1007.8	56,254	1289.0
75–79	33,263	1073.6	53,253	1385.7
80–84	24,853	1157.5	43,157	1397.0
85–89	15,123	1257.4	30,214	1341.7
> = 90	5,583	1065.9	15,486	952.1

**Fig. 1:**
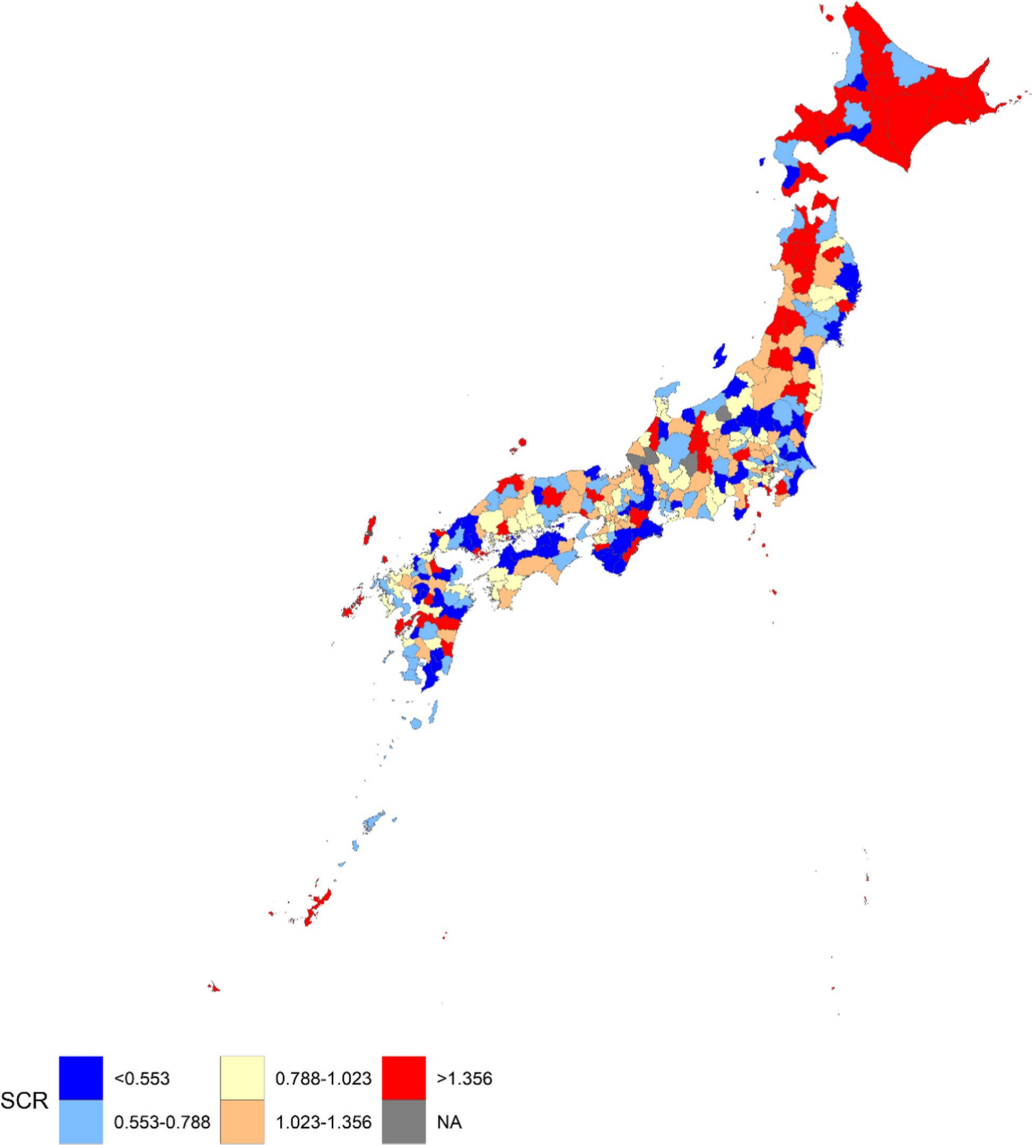
Geographic difference in SCR for the multi-psychotropic drug prescription in Japan. SCR, standardized claim ratio. Secondary medical areas were classified into quintiles based on value of the SCR

Figure 1 illustrates the geographic difference in SCR for the multi-psychotropic drug prescription in Japan. Median of the SCR was 0.88 and the mean was 1.03. Therefore, the SCR distribution was right-skewed. The higher altitudes of Japan, including Hokkaido, Aomori, and Akita prefectures, were typically where the high SCRs were found. Particularly, areas in Hokkaido, which are located at the north end of Japan, tended to have the highest-level SCR.

**Fig. 2:**
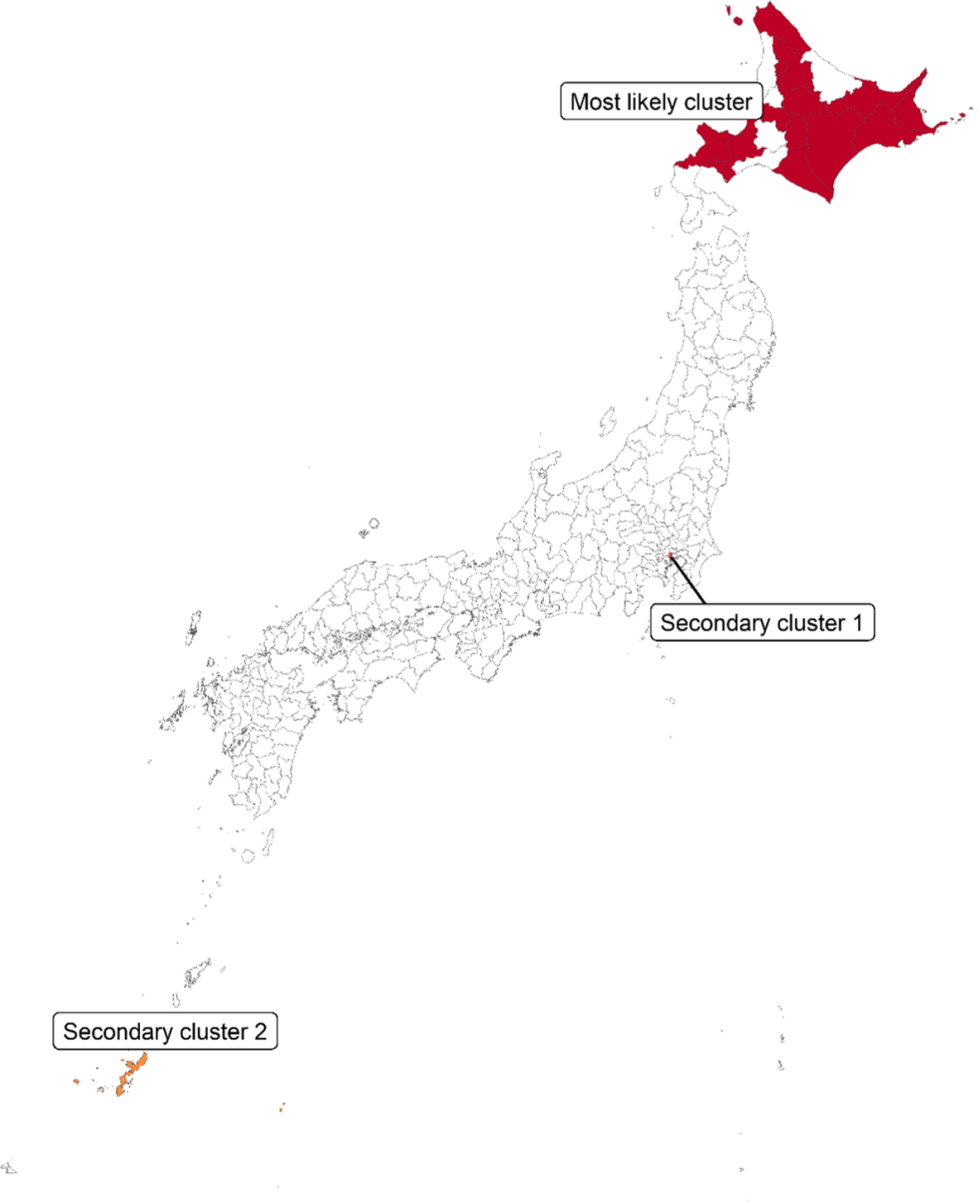
The most likely cluster, the secondary cluster 1, and the secondary cluster 2, which were identified by a spatial cluster detection method

Figure 2 depicts the most likely cluster identified by a spatial cluster detection method. 13 areas in Hokkaido were detected as the most likely cluster for the multi-psychotropic drug prescription, and the overall SCR was 1.902 (*p*-value = 0.001) in those areas. That is, an observed number of the multi-psychotropic drug prescription in those areas is 1.9 times higher compared with the expected value. Secondary clusters 1 and 2 were also detected in Tokyo and Okinawa, respectively. The SCR for the secondary cluster 1 was 2.767 (*p*-value = 0.001) and the overall SCR for secondary cluster 2 was 1.871 (*p*-value = 0.001).

Table 2 shows summary statistics for the areal features for the identified clusters and the other areas. The median population density was 0.4 person per hectare and lower in the most likely cluster compared with the other areas. In the most likely cluster, the median proportion of single-person households and the proportion of people with a lower educational level were 39.1% and 17.5%, respectively, which were higher than those in the other areas. The number of physicians per 100,000 people and the number of physicians working in a psychiatric department per 100,000 people in secondary cluster 1 were 1198.3 and 49.5, respectively, which were considerably higher than those in the other areas.

**Table 2:** Summary statistics for the areal features for the identified clusters and the other areas

Areal features	Most likely cluster (13 areas)	Secondary cluster 1 (one area)	Secondary cluster 2 (three areas)	The other areas
	Median (IQR)	Value	Median (IQR)	Median (IQR)
Population	182,510 (66,894–230,748)	904,344	516,417 (309,569–631,020)	245,102 (119,579–511,768)
Number of the multi-drug prescriptions	4115 (2137–5589)	31,875	8926 (5458–13,486)	2506 (1092–6050)
Total area (hectare)	423,810 (343,832–481,115)	6364	38,876 (37,834–54,711)	83,453 (43,811–135,412)
Population density (person per hectare)	0.4 (0.2–0.5)	142.1	14.0 (7.7–16.6)	2.7 (1.1–7.4)
Proportion of single-person households (%)	39.1 (38.4–40.8)	56.9	36.8 (36.6–39.2)	32.8 (29.5–36.1)
Proportion of divorced persons (%)	6.5 (5.9–6.9)	4	7.4 (7.2–7.8)	5.5 (4.8–6.3)
Proportion of persons with lower educational level (%)	17.5 (15.7–20.8)	3.3	12.9 (12.5–15.8)	13.4 (10.4–16.7)
Proportion of unemployed persons (%)	3.7 (3.0–4.3)	2.7	5.5 (5.4–5.7)	3.8 (3.5–4.2)
Total number of prescriptions per capita	6.0 (5.5–6.1)	12.8	5.2 (5.0–5.5)	6.0 (5.2–6.6)
Number of physicians ^a^	179.4 (131.7–212.0)	1198.3	207.0 (200.4–258.5)	194.8 (165.2–249.7)
Number of physicians working in a psychiatric department ^a^	10.5 (9.1–16.8)	49.5	20.3 (16.5–23.0)	11.8 (9.3–16.5)
Number of medical institutions with a psychiatric department ^a^	6.5 (4.8–7.2)	38.8	10.7 (8.7–10.7)	6.7 (5.2–8.8)

*IQR* Interquartile range, *SCR* Standardized claim ratio

^a^ Number per 100,000 persons

Table 3 shows the results of the correlation coefficient between the SCR and the areal features. The result indicates bivariate correlation between the SCR and each of the areal features and does not indicate the multivariate analysis result. There was a statistically significant positive correlation between the SCR and the proportion of single-person households (*p*-value = 0.014), the total number of prescriptions against per capita (*p*-value < 0.001), the number of physicians per capita (*p*-value = 0.016), the number of medical institutions with a psychiatric department per capita (*p*-value < 0.001), and the number of physicians working in a psychiatric department per capita (*p*-value < 0.001). Particularly, the correlations with the medical characteristics were high.

**Table 3:** Results of the correlation coefficient between the SCR and the areal features

Areal features	Correlation coefficient	*p*-value
Population density (person per hectare)	0.007	0.899
Proportion of single-person households (%)	0.138	0.014
Proportion of divorced persons (%)	0.087	0.124
Proportion of persons with lower educational level (%)	0.101	0.074
Proportion of unemployed persons (%)	0.003	0.963
Total number of prescriptions per capita	0.244	< 0.001
Number of physicians ^a^	0.135	0.016
Number of physicians working in a psychiatric department ^a^	0.204	< 0.001
Number of medical institutions with a psychiatric department ^a^	0.203	< 0.001

^a^ Number per 100,000 persons

Table 4 displays the results of spatial multivariate regression analysis investigating factors related to the SCR. The SCR was statistically positively associated with the percentage of people with lower educational levels (*p*-value = 0.006), total number of prescriptions per capita (*p*-value < 0.001), and the number of physicians working in a psychiatric department per capita (*p*-value = 0.025).

**Table 4:** Results of spatial regression analysis investigating factors associated with the SCR

Areal features	SPRC (95% CI)	*p*-value
Population density (person per hectare)	− 0.095 (− 0.260, 0.071)	0.264
Proportion of single-person households (%)	0.148 (− 0.004, 0.300)	0.056
Proportion of divorced persons (%)	− 0.153 (− 0.326, 0.020)	0.083
Proportion of persons with lower educational level (%)	0.208 (0.060, 0.355)	0.006
Proportion of unemployed persons (%)	0.032 (− 0.090, 0.155)	0.607
Total number of prescriptions per capita	0.228 (0.107, 0.349)	< 0.001
Number of physicians ^a^	− 0.086 (− 0.239, 0.067)	0.269
Number of physicians working in a psychiatric department ^a^	0.161 (0.020, 0.301)	0.025
Number of medical institutions with a psychiatric department ^a^	0.122 (− 0.015, 0.259)	0.080

*SPRC* Standardized partial regression coefficient, *CI* Confidence interval

^a^ Number per 100,000 persons

## Discussion

This study examined regional differences in SCR for multi-drug prescriptions of psychotropic drugs in Japan and its associated factors. As a result, it was discovered that Hokkaido contains a collection of high-SCR areas. Moreover, a spatial analysis identified some spatial clusters of multi-psychotropic drug prescriptions, and values for key regional characteristics were considerably different among the spatial clusters. It was suggested that multi-psychotropic drug prescriptions are a multifactorial problem, with the weight of each cause varies depending on the geographical region. Furthermore, the proportion of persons with lower educational levels was statistically positively associated with SCR. Studies from other nations have found a link between multiple drug use and a lower educational level [40–43], but to our knowledge, this study is the first study that investigated the association in Japan. We discuss possible reasons for the association.

It was discovered that there were many areas where the SCR was high, especially in Hokkaido, and some areas in Aomori and Akita prefectures were also high. It is known that the prescription amount of hypnotics per capita is high in Hokkaido and that anxiolytics are high in Hokkaido, Aomori, and Akita [14]. Contrarily, it is untrue that all of Hokkaido’s regions have high SCRs; regional variations in SCR can also be found within prefectures. The numbers related to medical characteristics per capita were not higher in the most likely cluster compared with the other areas. In contrast, the proportion of single-person households and the proportion of persons with lower educational level, which were positively correlated or associated with the SCR, were higher in the most likely cluster compared with the other areas. These factors might be a reason for the high SCRs in those areas.

The number of physicians working in a psychiatric department per capita was positively associated with SCR. Psychiatrists frequently prescribe psychotropic drugs [44], and it is thought that areas with a high concentration of psychiatrists tend to have more prescriptions for psychotropic drugs. Although the reimbursement fees decrease against multi-psychotropic drug prescriptions, prescriptions were not applied when antipsychotics or antidepressants are unavoidably prescribed by a doctor with sufficient training in psychiatric clinical practice. However, anxiolytics and hypnotics are exceptions. In addition, the reimbursement fees reduction occurs when multi-psychotropic drugs are prescribed by psychiatrists without sufficient clinical experience. Therefore, it is still possible that some of the reimbursement fees reduction were caused by psychiatrists. In addition, psychiatrists may tend to conduct multi-psychotropic drug prescriptions because an association between psychiatrists and multi-psychotropic drug prescription or polypharmacy are shown in other countries [45–47]. Moreover, it is mentioned in a previous study that factors, such as physician's culture, patient's attitude, and lack of training in psychopharmacology are possible reasons why psychiatrists in Japan continue to prescribe multiple drugs and high doses of psychotropics compared with other countries [1].

The number of medical institutions with a psychiatric department per capita and the number of physicians working in a psychiatric department per capita were also correlated with the SCR. These correlation might be observed due to a positive association between the SCR and the number of physicians working in a psychiatric department per capita. Additionally, the total number of prescriptions per capita was also positively associated with the SCR. It is natural that the number of multi-psychotropic drug prescriptions is high in areas where the total number of prescriptions is high.

As a possible reason for the association between the proportion of persons with lower educational levels and SCR, it is thought that the prevalence of people with a psychiatric disease is high in those regions. In Japan, it is well known that psychological distress and depressive symptoms have a statistically significant positive correlation with lower income or lower educational attainment [48, 49]. Furthermore, polypharmacy and lower educational attainment may be positively correlated in Japan, while the association has not been investigated yet. In other countries, it has been confirmed that subjects with low education had a higher probability of polypharmacy, inappropriate drug use, or using three or more psychotropic drugs [40–43]. In Spain, educational attainment was associated with medication literacy [50], and inappropriate drug use may occur in people with lower educational levels. As another possibility, the treatment rate of a psychiatric disease may be high in persons with lower educational levels as the reason for the association shown in the results. It is known that the treatment rate among psychologically distressed people in Japan is relatively low in people with high incomes or full-time workers [51], while high socioeconomic status is rather positively associated with mental health treatment among countries in the world [52].

As a result of this study, it is critical to reexamine whether inappropriate multi-drug prescriptions tend to exist or not in regions with particularly high SCR. In addition, it is believed that SCR is positively correlated with the prevalence of patients with psychiatric diseases in the region, and administrative measures to reduce the prevalence in high-SCR regions are also required. Furthermore, future research into the mechanism for the association between SCR and the proportion of persons with lower educational levels among regions is needed in the future. It is particularly important to investigate the relationship between lower educational levels and multi-drug use of psychotropic drugs in Japan using individual data. If people with lower educational levels do tend to have polypharmacy for psychotropic drugs, an education about negative effects of multi-psychotropic drug use against patients with lower educational levels are required in communities or medical facilities.

This research has some limitations. First, this study used the number of multi-drug prescriptions for psychotropic drugs as the outcome, but the number of patients with psychotropic polypharmacy is not publicly available. There is a possibility that a small number of patients received several multi-drug prescriptions or that a larger number of patients received fewer multi-drug prescriptions, and the number of multi-drug prescriptions per one patient might differ depending on geographical region. However, the multi-drug prescription is against policy recommendations in Japan, and a reduction in the prescriptions is needed in either case. In addition, data on prescribed dose for each of secondary medical area were not publicly available. It will be meaningful to survey average daily dose or total dose of prescribed psychiatric drugs for each area in the future. Second, this is an ecological study, and an ecological fallacy might exist in the association. It will be meaningful to examine the predictors of patients with psychotropic polypharmacy using individual-level data. Third, we were unable to obtain data by gender or region, and data on the number of multi-drug prescriptions for each type of psychotropic drug were not available. Fourth, data on in-hospital prescriptions were not used in this study because there were many areas where the number of in-hospital prescriptions for multi-drug psychotropic drugs was not available. In general, prescriptions for hospitalized patients are included in in-hospital prescriptions. An analysis including data on the in-hospital prescriptions is desirable in the future. However, the number of in-hospital prescriptions for multi-psychotropic drugs was one-seventh of that of out-of-hospital prescriptions [18], and it was relatively lower compared to out-of-hospital prescriptions. Fifth, as stated in the Methods, the prevalence of electronic receipts is over 95% in Japan, but there may be some secondary medical areas where the prevalence of electronic receipts is relatively low compared to other areas. The prevalence of electronic receipts for each secondary medical area was not publicly available, which is also a study limitation. Sixth, the causes of multi-psychotropic drug prescriptions could not be scrutinized. In some cases, the multi-psychotropic drug prescriptions might be caused by inappropriate prescriptions, but they are conducted for the severity of illnesses in other cases [53]. In addition, the current multi-psychotropic drug prescriptions might indicate the healthcare providers' delay to adapt to governmental policies because it is known that multi-drug prescriptions decreased due to policies over the recent years in Japan [54]. Moreover, the regional differences might be affected by rigor of “the Healthcare bill check and payment organization” because the number of multi- psychotropic drug prescriptions are based on self-reporting by medical institutions. Therefore, further studies focusing on causes and contents of the multi-psychotropic drug prescriptions is necessary in the future.

## Conclusions

We revealed regional differences in the multi-drug prescriptions of psychotropic drugs in Japan and detected factors associated with it using nationally representative data. As a result, it was discovered that a spatial cluster for the high rate of multi-psychotropic drug prescription exists in Hokkaido. Furthermore, a spatial regression analysis showed that the proportion of persons with lower educational levels, the total number of prescriptions per capita, and the number of physicians working in a psychiatric department per capita were statistically positively associated with the SCR for multi-psychotropic drug prescription.

## Data Availability

The data analysed during the current study are publicly available. Data on the Survey of population, demographics, and household number based on the Basic Resident Register were available from: https://www.e-stat.go.jp/stat-search/files?page=1&toukei=00200241&tstat=000001039591. Data on the Census were available from: https://www.e-stat.go.jp/stat-search/files?page=1&toukei=00200521. Data on the NDB Open data were available from: https://www.mhlw.go.jp/stf/seisakunitsuite/bunya/0000177182.html. Data on the State of prefectures and municipalities (System of social and demographic statistics) were available from: https://www.e-stat.go.jp/regional-statistics/ssdsview. Data on the Statistics of Physicians, Dentists, and Pharmacists were available from: https://www.e-stat.go.jp/stat-search/files?page=1&toukei=00450026&kikan=00450&result_page=1. Data on the Survey of Medical Institutions were available from: https://www.e-stat.go.jp/stat-search/files?page=1&toukei=00450021&tstat=000001030908. The digital national land information were available from: https://nlftp.mlit.go.jp/ksj/gml/datalist/KsjTmplt-N03-v3_0.html.

## Abbreviations

SPRC: Standardized partial regression coefficient
CI: Confidence intervals
SCR: Standardized claim ratio
NDB: National Database of Health Insurance Claims and Specific Health Checkups of Japan
